# Supplementary material for: Characterization of sound scattering layers in the Bay of Biscay using broadband acoustics, nets and video
Source: PLoS One. 2019 Oct 21;14(10):e0223618. doi: 10.1371/journal.pone.0223618 (PMC6802824; doi:10.1371/journal.pone.0223618)
Supplement: S1 Appendix — (PDF) [file pone.0223618.s001.pdf]

S1 Appendix: Echosounder calibrations

Several calibrations performed with 22mm and 38.1mm tungsten carbide (WC) with 6% cobalt binder spheres were necessary to obtain acceptable Root Mean Square Error (RMSE) for calibration gains (Table 1). Due to bad weather, the PELGAS 2016 calibration gain RMSEs were too high (higher than 0.4). Calibration gains obtained *in situ* during the PELGAS2017 survey were used for the 70 and 120 kHz echosounders. *Ex situ* calibrations of the 200 kHz and 333 kHz transducers were performed in June 2017 in the IFREMER tank in Brest, using a 22 mm sphere tethered with monofilament lines than were thiner than those used at sea (0.1 and 0.7 mm diameter, respectively). Calibration was performed using the SIMRAD EK80 software (version 1.11.1) with default calibration settings. The EK80 software did not compute the gain in the sphere nulls. The gains for these frequencies were obtained by a linear interpolation in order to fill these gaps. Calibration gains and RMSE spectra are presented in figure A.

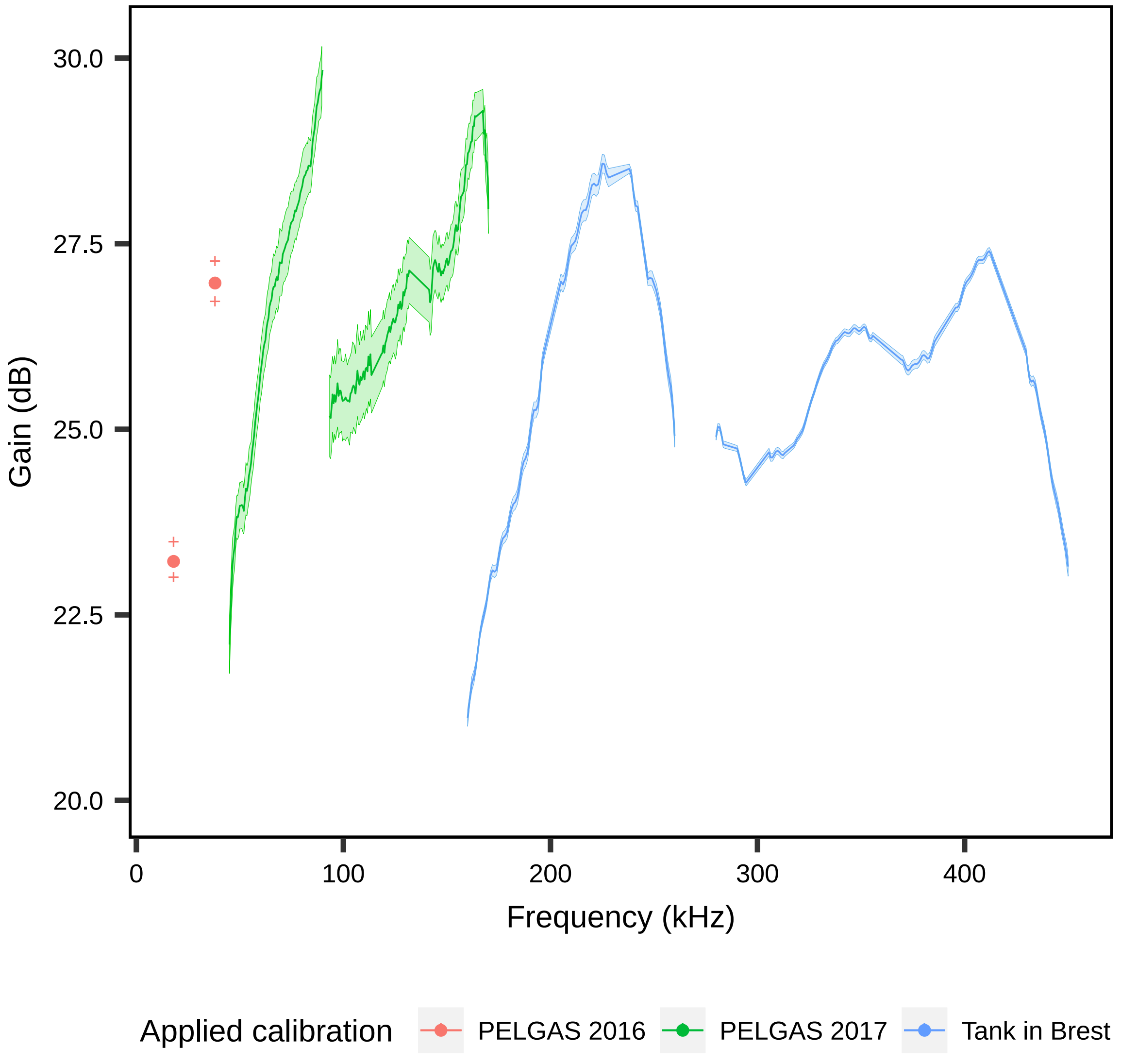

Figure A: Calibration gains (solid lines) and Root Mean Square Error (RMSE, shaded areas) for each echosounder as a function of acoustic frequency.
