# Supplementary material for: Characterization of sound scattering layers in the Bay of Biscay using broadband acoustics, nets and video
Source: PLoS One. 2019 Oct 21;14(10):e0223618. doi: 10.1371/journal.pone.0223618 (PMC6802824; doi:10.1371/journal.pone.0223618)
Supplement: S2 Appendix — (PDF) [file pone.0223618.s002.pdf]

S2 Appendix : Gaseous target backscattering model

We define the flattening factor ϵ as the ratio of half large and small axis of the prolate spheroid and the equivalent spherical radius a_{eq} with: $a_{eq} = a(\epsilon)^{-2/3}$.

The TS of gaseous target close to resonance can expressed, according to [1] and compatible with [2], and [3].

$$TS = 10\log_{10}\left(\frac{\chi^2 \times a_{eq}^2}{\left[(\frac{f_0}{f})^2 - 1 - 2\frac{\delta_0}{\omega}ka_{eq}\right]^2 + \left[2\frac{\delta_0}{\omega} + (\frac{f_0}{f})^2 ka_{eq}\right]^2}\right) \tag{1}$$

Where f is the frequency of the transmitted sound wave, f_0 is the resonance frequency, , δ_0 the damping factor other than radiative one and χ the correction factor of the spheroid flattening [4] [5], expressed by:

$$\chi = \epsilon^{-1/3}\left(\frac{\log(\frac{1+(1-\epsilon)^{1/2}}{\epsilon})}{(1-\epsilon)^{1/2}}\right)^{-1/2} \tag{2}$$

Resonant frequency f_0 of the bubble is expressed as a function of equivalent spherical radius a_{eq}, of the ratio of gas specific heat γ (1.4 for air), of the hydrostatic pressure P, of the surrounding medium density ρ, of the surface tension of the bubble τ [1] and of the correction factor χ :

$$f_0 = \frac{\chi}{2\pi a_{eq}} \times \left(\frac{3\gamma P + 2 * \frac{\tau}{a_{eq}} \times (3 \times \gamma - 1)}{\rho}\right)^{1/2} \tag{3}$$

The hydrostatic pressure P (en Pa) at depth d (en m) is express as:

$$P = (1 + 0.1d)10^5 \tag{4}$$

The damping factor δ_0 is express as:

$\delta_0 = \delta_{vis} + \delta_{th}$

With δ_{vis} the surrounding fluid viscosity damping factor and δ_{th} the gas thermal dissipation damping factor.

The viscosity damping δ_{vis} is defined by:

$$\delta_{vis} = \frac{2\eta_s}{\rho\omega a_{eq}^2} \tag{5}$$

with η_s the dynamic viscosity of the fluid surrounding the bubble, that express the fluid resistance to the shearing stress generated by the oscillation of the bubble around its balanced position.

The thermal damping δ_{th} is defined by:

$$\delta_{th} = \frac{3P_{gas}}{\rho\omega^2 a_{eq}^2} Im(\Gamma) \tag{6}$$

With P_{gaz} the balance gas pressure as a function of hydrostatic pressure P of surrounding fluid and the surface tension τ:

$$P_{gaz} = P + \frac{2\tau}{a_{eq}} \tag{7}$$

and the adiabatic complex coefficient :

$$\Gamma = \frac{\gamma}{1 - \left[\frac{(1+i)X/2}{\tanh[(1+i)X/2]} - 1\right] \frac{6i(\gamma-1)}{X^2}} \tag{8}$$

Where X is the rapport of thermal diffusion:

$$X = \frac{a_{eq}}{l_{th}} \tag{9}$$

With l_{th}, the specific length of heat diffusion link to the diffusivity D by :

$$l_{th} = \sqrt{\frac{D}{2\omega}} \tag{10}$$

With in particular the air thermal diffusivity : $D_{air} = 1,9.10^{-5}(m^2/s)$.

References

1. Ainslie MA, Leighton TG. Review of scattering and extinction cross-sections, damping factors, and resonance frequencies of a spherical gas bubble. The Journal of the Acoustical Society of America. 2011;130(5):3184–3208. doi:10.1121/1.3628321.

2. Andreeva I. Scattering of sound by air bladders of fish in deep sound-scattering ocean layers. Sov Phys Acoust. 1964;10:17–20.

3. Weston DE. Sound propagation in the presence of bladder fish. Underwater acoustics. 1967;2:55–88.

4. Ye Z. Low-frequency acoustic scattering by gas-filled prolate spheroids in liquids. The Journal of the Acoustical Society of America. 1997;101(4):1945–1952. doi:10.1121/1.418225.

5. Love RH. Resonant acoustic scattering by swimbladder-bearing fish. The Journal of the Acoustical Society of America. 1978;64(2):571. doi:10.1121/1.382009.
