# Supplementary figures and images for: Characterization of sound scattering layers in the Bay of Biscay using broadband acoustics, nets and video
Source: PLoS One. 2019 Oct 21;14(10):e0223618. doi: 10.1371/journal.pone.0223618 (PMC6802824; doi:10.1371/journal.pone.0223618)

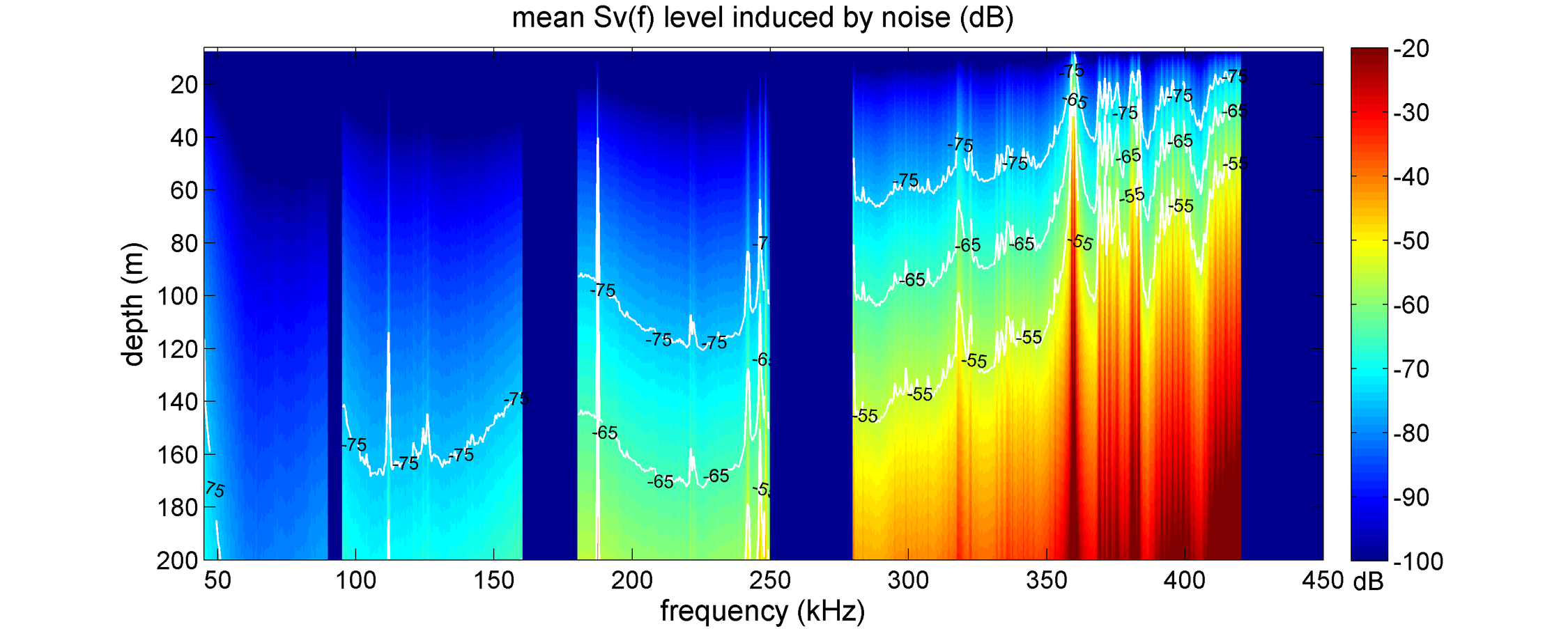

Supplement: S1 Fig — Noise measurements (volume backscattering in decibel, color key) performed with the echosounders in passive mode during the PELGAS2017 survey. x-axis: acoustic frequency (kHz), y-axis: range from transducer (m). (TIF) [file pone.0223618.s003.tif]

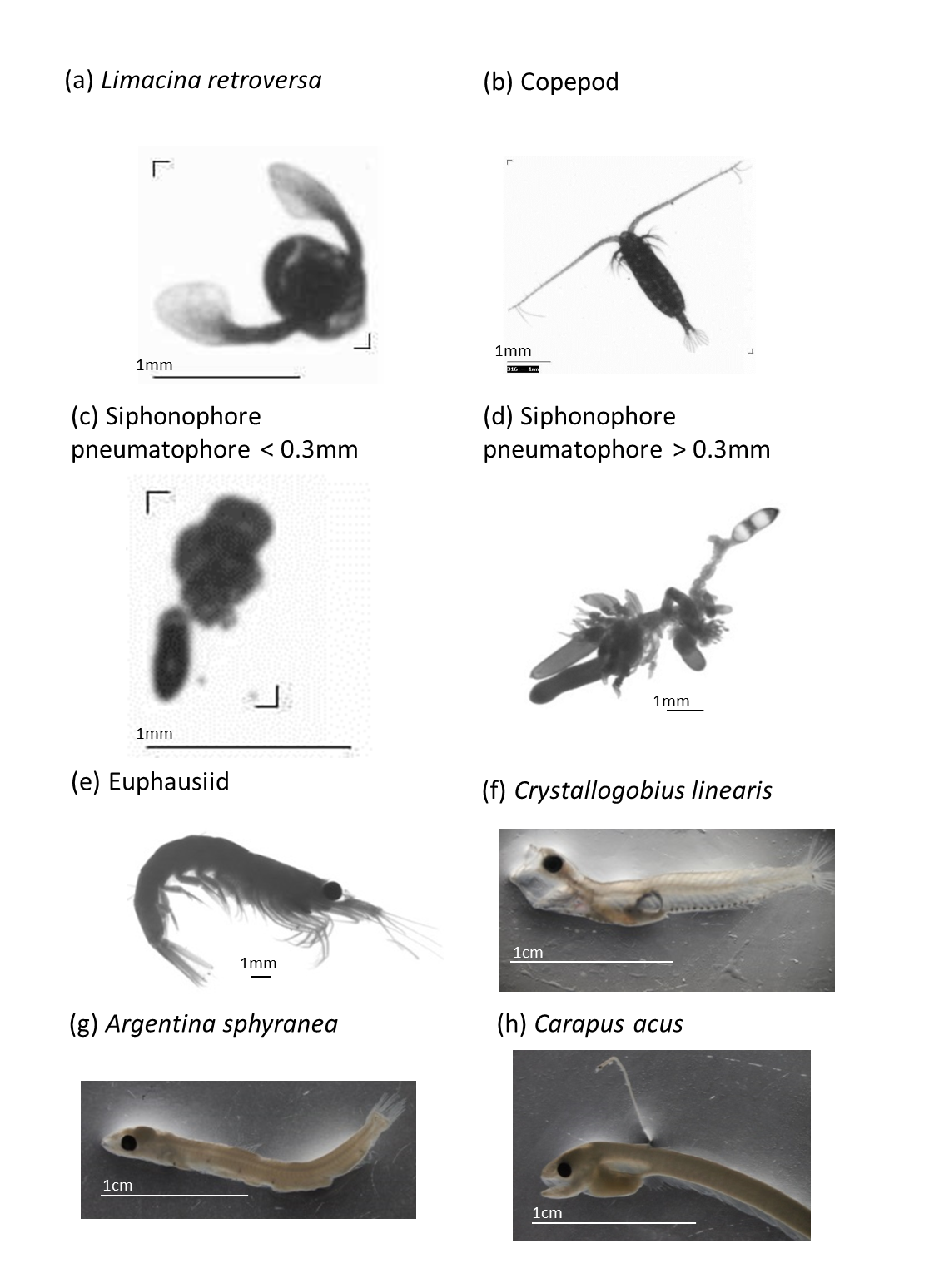

Supplement: S2 Fig — Images of main scatterers in the samples taken with (a), (b) and (c): zooCAM; (d) and (e): Zooscan; (f), (g) and (h): binocular microscope. (TIF) [file pone.0223618.s004.tif]

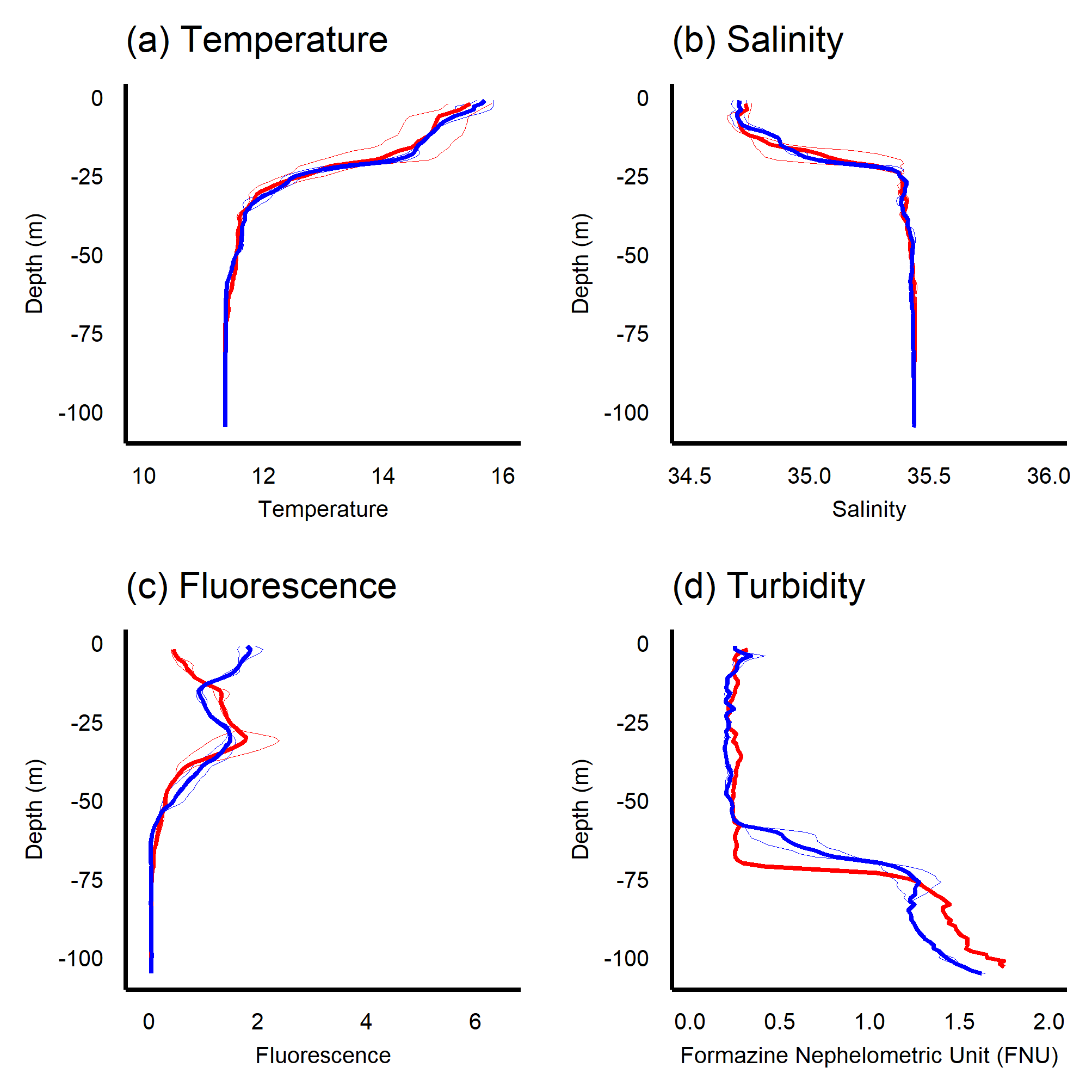

Supplement: S3 Fig — Temperature (a), salinity (b), fluorescence (c) and turbidity (d) profiles. Each dashed line represent one profile, the solid lines represent the mean profile for daytime (red) and night time (blue). (TIF) [file pone.0223618.s005.tif]
