# Supplementary material for: Characterization of sound scattering layers in the Bay of Biscay using broadband acoustics, nets and video
Source: PLoS One. 2019 Oct 21;14(10):e0223618. doi: 10.1371/journal.pone.0223618 (PMC6802824; doi:10.1371/journal.pone.0223618)
Supplement: S1 Table — Depth and time of the net tows. Each gear was associated with a station number. The multinet open each of its nets at a specified depth, presented in net depth interval. Only the multinet nets sampling in a sampled layer are presented in this table. (PDF) [file pone.0223618.s006.pdf]

S4 Table : Description of used mesozooplankton and micronekton nets

Table 1. Depth and time of the net tows. Each gear was associated with a station number. The multinet open each of its nets at a specified depth, presented in net depth interval. Only the multinet nets sampling in a sampled layer are presented in this table.

Station number	Net type	Net depth interval (m) (multinet)	sampled layer	Max depth (m)	Mean depth (m)	Tow duration (min)	UTC times
U0337	MIK		surface layer	12.1	9.5	23	11:55 27/05/2016
U0339	MIK		deep layer	97	64	31	13:32 27/05/2016
U0341	Multinet	90-80	deep layer	90	85.3	6	15:00 27/05/2016
U0341	Multinet	30-10	surface layer	30	20.1	3	15:00 27/05/2016
U0352	MIK		deep layer	95	64	28:30	15:04 28/05/2016
U0353	MIK		surface layer	12	9	14	15:48 28/05/2016
U0354	Multinet	100-80	deep layer	100	93	10:30	16:31 28/05/2016
U0354	Multinet	30-10	surface layer	30	22.7	13:30	16:31 28/05/2016
