# Supplementary material for: Characterization of sound scattering layers in the Bay of Biscay using broadband acoustics, nets and video
Source: PLoS One. 2019 Oct 21;14(10):e0223618. doi: 10.1371/journal.pone.0223618 (PMC6802824; doi:10.1371/journal.pone.0223618)
Supplement: Attachment — Submitted filename: PONE-D-19-17373 - Review Comments.pdf [file pone.0223618.s007.pdf]

This is one of a few high quality manuscripts I have reviewed over decades of my professional career. The field experiment was carefully designed and the co-registered acoustic, biological, optical, and oceanographic data were collected properly. Analysis of collected data including the scattering modeling and uncertainty assessment is scientifically sound and very objective. I recommend it be published with some minor revisions suggested below.

(Dezhang Chu)

1. Line 77: change “cover” to “covered”.
2. Lines 87-88: don’t know how this “horizontal distance of 10-12m” is derived. The ping rate is “one ping every 3 sec” (line 65) and the ship speed is “between 2 and 3 knots” (line 58), the horizontal distance should be 15 and 23 m, shouldn’t it?
3. Table 3: copepods g and h values seemed too large. The reference cited here (Lavery et al, 2007) is not a proper reference. These values provided in that paper were based on the other sources, one was a personal communication (with this reviewer) and the other was also a paper, of which the reviewer is the lead author, where the h was measured on decapod shrimp, not on copepods. This reviewer suggests to use lower values of g and h reported in another paper (Chu and Wiebe, 2005, ICES JMS 62:818-831). Although these new values will alter the results in Fig. 4, it won’t change the conclusion.
4. Lines 163, 320, 341: the symbol “>” or “>=” is wrong (an upside down question mark), possibly due to file conversion to pdf.
5. Equation (4): “bandwidth” in the denominator should be “number of frequency bins within the bandwidth”, shouldn’t it?
6. Table 5: copepod surface layer number density is “1403” but is “5300” on line 249, they are inconsistent.
7. Line 280 and Fig 3b: for cluster 2 (green line in Fig. 3b), it is a “decrease” not “increase” from 18 to 38 kHz. A typo?
8. Line 303: remove “Yet”.
9. Line 313: add “was” after “sampling”.
10. Line 317: add “commonly” after “frequencies”.
11. Line 324: change “little” to “mild”.
12. Line 330: remove “no”
13. Line 367: the symbol “<” or “<=” is wrong (an upside down exclamation mark), possibly due to file conversion to a pdf file.
14. Line 399: replace “belonging to” with “of”.
15. Line 400: (a) add an “a” before perspective; (b) remove “the” before “acoustic”.
16. Line 401: change “the general” to “a general”.
17. Line 402: add “climate” after “global”.
18. Figure 2: the blue boundary lines of the surface sampled layer are too hard to see. Please use other color (orange, or pink?) to make them easier to see.
19. Figure 3: the black trawl or net traces are superimposed on a purple color background and are also really hard to see, please change to a different color (red, or pink?).

20. Figures 2- 4: the resolution is too low and all the x-axis and y-axis labels are blurred. Please use a higher resolution for these figures.
