# Supplementary material for: Characterization of sound scattering layers in the Bay of Biscay using broadband acoustics, nets and video
Source: PLoS One. 2019 Oct 21;14(10):e0223618. doi: 10.1371/journal.pone.0223618 (PMC6802824; doi:10.1371/journal.pone.0223618)
Supplement: Attachment — Submitted filename: Response to reviewers PLOES ONE.docx [file pone.0223618.s008.docx]

| **Academic Editor** | **How did we address comments** |
| --- | --- |
| Please ensure that your manuscript meets PLOS ONE's style requirements, including those for file naming. The PLOS ONE style templates can be found at http://www.journals.plos.org/plosone/s/file?id=wjVg/PLOSOne_formatting_sample_main_body.pdf and http://www.journals.plos.org/plosone/s/file?id=ba62/PLOSOne_formatting_sample_title_authors_affiliations.pdf | Done |
| We note that you have stated that you will provide repository information for your data at acceptance. Should your manuscript be accepted for publication, we will hold it until you provide the relevant accession numbers or DOIs necessary to access your data. If you wish to make changes to your Data Availability statement, please describe these changes in your cover letter and we will update your Data Availability statement to reflect the information you provide. | The data analysed in this paper will soon be available on the Ifremer SEANOE (<https://www.seanoe.org/>) platform, with a DOI. |
| We note that you have included the phrase “data not shown” in your manuscript. Unfortunately, this does not meet our data sharing requirements. PLOS does not permit references to inaccessible data. We require that authors provide all relevant data within the paper, Supporting Information files, or in an acceptable, public repository. Please add a citation to support this phrase or upload the data that corresponds with these findings to a stable repository (such as Figshare or Dryad) and provide and URLs, DOIs, or accession numbers that may be used to access these data. Or, if the data are not a core part of the research being presented in your study, we ask that you remove the phrase that refers to these data. | We removed this phrase and the minor statement that it support. |
| Thank you for stating the following in the Acknowledgments Section of your manuscript:  A. Blanluet doctoral grant was co-funded by the Conseil Régional des Pays de la Loire and IFREMER.  We note that you have provided funding information that is not currently declared in your Funding Statement. However, funding information should not appear in the Acknowledgments section or other areas of your manuscript. We will only publish funding information present in the Funding Statement section of the online submission form.  Please remove any funding-related text from the manuscript and let us know how you would like to update your Funding Statement. Currently, your Funding Statement reads as follows:  The author(s) received no specific funding for this work. | We removed this statement from the acknowledgments Section, and we would ad it to our funding Statement. |

Reviewer 1

| **Comments of reviewer #1** | **How did we address comments** |
| --- | --- |
| First of all, for question 3 above I found some 2013 PELGAS data (475 MB) at  DORAY Mathieu, DUHAMEL Erwan, HURET Martin, PETITGAS Pierre, MASSE Jacques (2000) PELGAS, https://doi.org/10.18142/18,  but I never located the 2016 data analyzed in this paper. | The data analysed in this paper will soon be available on the Ifremer SEANOE (<https://www.seanoe.org/>) platform. |
| There is a font problem with greater than and/or less than symbols, as at line 11, line 163, line 341, line 367, maybe other places. | Corrected |
| The sentence at line 15 could be changed to begin 'Uncertainty of the species composition in the SSL's ... | Corrected |
| The resonance discussion (line 39) should mention that there is a frequency of energetic response related to the vibrational characteristics of the scatterer, the resonant frequency. The term 'given frequency' doesn't really convey any useful information. Also resonance is not restricted to gaseous inclusions. | We have corrected our discussion:  Line 40 : ''Resonance occurs when some specific types of targets (as gaseous inclusions) increasingly vibrate upon insonification by a narrow frequency band [25, 26], given by the target size and vibrational characteristics. This resonance can induce a dramatic increase in the backscattering intensity of small gas-bearing organisms (e.g. physonect siphonophores or small swimbladdered fish), relatively to their size [4, 14].'' |
| Line 104: Do you mean fluorometer? | Corrected |
| Page 7: I could not find what EI means. | The initials EI (for “echo-integration”) have been substituted by the term “echo-integration” to reduce ambiguity |
| page 11: I could not determine the reason for making Sv standardized. Is this a function of frequency? If Sv(f) is from calibrated systems, what is the reason for adding on the quantity that is an average over the transducers? (N=6 transducers, correct?) | The objective of the standardisation prior to the clustering was to be able to compare exclusively the frequency spectra by dividing all spectra by their means Sv. In order to not give a higher importance at the high frequencies (with more frequencies point that low frequencies), we weighed all channel by their bandwidth (equation 4). |
| The language of the sentence at line 364 needs repair. I don't understand the statement. This is true at line 414 also, in S1 Appendix. | Line 365: “The uncertainty analysis enabled to represent the potential errors on model parameters and better assess the discrepancies between predicted and measured Sv(f) curves.”  Line 414: “The EK80 software did not compute the gain in the sphere nulls. The gains for these frequencies were obtained by a linear interpolation in order to fill these gaps.” |

Reviewer 2

| **Comments of reviewer #2** | **How did we address comments** |
| --- | --- |
| Line 77: change “cover” to “covered”. | corrected |
| Lines 87-88: don’t know how this “horizontal distance of 10-12m” is derived. The ping rate is “one ping every 3 sec” (line 65) and the ship speed is “between 2 and 3 knots” (line 58), the horizontal distance should be 15 and 23 m, shouldn’t it? | Indeed, the 10-12m distance derived from an old echo-integration. We corrected this distance. |
| Table 3: copepods g and h values seemed too large. The reference cited here (Lavery et al, 2007) is not a proper reference. These values provided in that paper were based on the other sources, one was a personal communication (with this reviewer) and the other was also a paper, of which the reviewer is the lead author, where the h was measured on decapod shrimp, not on copepods. This reviewer suggests to use lower values of g and h reported in another paper (Chu and Wiebe, 2005, ICES JMS 62:818-831). Although these new values will alter the results in Fig. 4, it won’t change the conclusion. | We corrected our values for the ones suggested by the reviewer |
| Lines 163, 320, 341: the symbol “>” or “>=” is wrong (an upside down question mark), possibly due to file conversion to pdf. | corrected |
| Equation (4): “bandwidth” in the denominator should be “number of frequency bins within the bandwidth”, shouldn’t it? | corrected |
| Table 5: copepod surface layer number density is “1403” but is “5300” on line 249, they are inconsistent. | Corrected, the “5300 ind/m^3^“ were the remaining night density of Limacina from a previous version of the manuscript |
| Line 280 and Fig 3b: for cluster 2 (green line in Fig. 3b), it is a “decrease” not “increase” from 18 to 38 kHz. A typo? | This was an unclear phrasing, we repair the sentence : (line 280) “The median Sv(f) spectrum of each cluster displayed specific shapes: a peak at 38 kHz then a rise for cluster 3; a flat spectrum for cluster 2 and a bump between 18 and 150 kHz for the cluster 1, followed by a flat curve beyond 150 kHz.” |
| Line 303: remove “Yet”. | corrected |
| Line 313: add “was” after “sampling”. | corrected |
| Line 317: add “commonly” after “frequencies”. | corrected |
| Line 324: change “little” to “mild”. | corrected |
| Line 330: remove “no” | corrected |
| Line 367: the symbol “<” or “<=” is wrong (an upside down exclamation mark), possibly due to file conversion to a pdf file. | corrected |
| Line 399: replace “belonging to” with “of”. | corrected |
| Line 400: (a) add an “a” before perspective; (b) remove “the” before “acoustic”. | corrected |
| Line 401: change “the general” to “a general”. | corrected |
| Line 402: add “climate” after “global”. | corrected |
| Figure 2: the blue boundary lines of the surface sampled layer are too hard to see. Please use other color (orange, or pink?) to make them easier to see. | corrected |
| Figure 3: the black trawl or net traces are superimposed on a purple color background and are also really hard to see, please change to a different color (red, or pink?). | The difficulty to see the black line were due to the low resolution of the figure. We thickened the net trace to be seen easily. |
| Figures 2- 4: the resolution is too low and all the x-axis and y-axis labels are blurred. Please use a higher resolution for these figures. | The low resolution is due to the revue figure compression, for the pdf. The figure will be at a better resolution for the final version of the paper. |
